# Children with developmental coordination disorder are less able to fine-tune muscle activity in anticipation of postural perturbations than typically developing counterparts

**DOI:** 10.3389/fnhum.2023.1267424

**Published:** 2023-10-27

**Authors:** Carla Harkness-Armstrong, Emma F. Hodson-Tole, Greg Wood, Richard Mills

**Affiliations:** ^1^Centre for Physical Activity in Health and Disease, Division of Sport, Health, and Exercise Sciences, Brunel University London, Uxbridge, United Kingdom; ^2^Department of Life Sciences, Manchester Metropolitan University, Manchester, United Kingdom; ^3^Manchester Metropolitan University Institute of Sport, Manchester, United Kingdom; ^4^Department of Sport and Exercise Sciences, Manchester Metropolitan University, Manchester, United Kingdom

**Keywords:** balance, postural control, dyspraxia, electromyography, motor control, entropy halflife

## Abstract

The majority of children with developmental coordination disorder (DCD) struggle with static and dynamic balance, yet there is limited understanding of the underlying neuromechanical mechanisms that underpin poor balance control in these children. Eighteen children with DCD and seven typically developing (TD) children aged 7–10 years stood with eyes open on a moveable platform progressively translated antero-posteriorly through three frequencies (0.1, 0.25 and 0.5 Hz). Myoelectric activity of eight leg muscles, whole-body 3D kinematics and centre of pressure were recorded. At each frequency, postural data were divided into transition-state and steady-state cycles. Data were analyzed using a linear mixed model with follow-up Tukey’s pairwise comparisons. At the slowest frequency, children with DCD behaved like age-matched TD controls. At the fastest frequency, children with DCD took a greater number of steps, had a greater centre of mass variability, had a greater centre of pressure area, and tended to activate their muscles earlier and for longer than TD children. Children with DCD did not alter their postural response following prolonged exposure to platform movement, however they made more, non-structured postural adjustments in the medio-lateral direction as task difficulty increased. At the faster oscillation frequencies, children with DCD adopted a different muscle recruitment strategy to TD children. Activating their muscles earlier and for longer may suggest that children with DCD attempt to predict and react to postural disturbances, however the resulting anticipatory muscle excitation patterns do not seem as finely tuned to the perturbation as those demonstrated by TD children. Future work should examine the impact of balance training interventions on the muscle recruitment strategies of children with DCD, to ensure optimal interventions can be prescribed.

## 1. Introduction

Developmental coordination disorder (DCD) is a movement disorder characterized by reduced motor competence and poor motor coordination, in the absence of other identifiable neurological and/or medical disorders (American Psychiatric Association, 2013). Affecting 5–6% of school-aged children (Zwicker et al., 2012), children with DCD experience significant problems in their fine and/or gross motor skills (Geuze et al., 2001). Most children with DCD also experience significant difficulties with both static and dynamic balance, which can lead to secondary issues such as non-participation in physical activity (Fong et al., 2011) and an increased risk of tripping and falling (Scott-Roberts and Purcell, 2018). As balance is integral in the successful performance of most functional skills (Huxham et al., 2001), it is essential to study the underlying mechanisms that may underpin poor balance control in children with DCD, to ensure that optimal interventions can be prescribed.

It is well established that, even for highly repetitive or simple balance tasks, human movement patterns are varied (Hausdorff, 2007; Turnock and Layne, 2010). However, this variation is not random, with patterns that can be quantified evident in the changes that occur. This time-based organization of variation, or structure, in movement patterns is recognized as an important feature of a neuromuscular system that can adapt to perturbations and changes in the surrounding environment (Bolton, 2015). Variation in walking characteristics of typically developing (TD) children (age 3–14 years) is less structured (more random) than those of adults (Hausdorff et al., 1999). Therefore, studying structure within movement patterns can reveal variations in the growth and maturation of the motor control system. Structure also exists in the muscle activation and coordination that drives movements (Hodson-Tole and Wakeling, 2017; Wakeling and Hodson-Tole, 2018). These structures can change in response to postural control challenges (Ferrari et al., 2020), highlighting the importance of neuromuscular drive in determining motor behaviors.

Postural control can be distinguished into reactive (feedback) or anticipatory (feedforward) responses, whereby postural adjustments are either made subsequent, or prior, to a balance perturbation. Responses to postural disturbances also scale to the level of postural threat (Adkin et al., 2000) and depend of the size of the perturbation. For instance, during smaller perturbations, an ankle strategy is often effective, whereby torque generated about the ankle joint is sufficient to maintain balance (Massion, 1994). In larger perturbations, a more severe response may be required, such as a hip strategy, whereby large, rapid movements are generated about the hips to regain centre of mass (COM) equilibrium (Horak and Nashner, 1986). As we develop across the lifespan, we learn to adapt to different perturbations through mechanisms that are dynamic and flexible (Haddad et al., 2013). However, individuals with DCD often present with a poor organization of body movements in relation to the global environment (Green and Payne, 2018), therefore it is important to assess the postural responses of those with DCD during balance perturbations.

Reactive and anticipatory mechanisms of postural control have been described previously for single discrete perturbations in children with DCD. During unexpected perturbations, Cheng et al. (2018) found that children with DCD reacted later than TD children to a forward push, whereas Fong et al. (2015) reported no group differences when reacting to a backward moving platform. During planned movements, children with DCD presented with fewer anticipatory muscle activations when kicking a ball and climbing stairs (Kane and Barden, 2012), and had a shorter duration between muscle activity onset time and peak activation than TD children during a *Y*-balance test, which was suggested to be a potential mechanism to compensate for a less-effective feedforward control system (Yam and Fong, 2019). Whilst knowledge of postural control during single perturbations is important, it is also essential to assess movement strategies during continuous dynamic situations (such as a moving base of support), to fully understand the underlying mechanisms that may contribute to poor balance control (Horak et al., 2009). The oscillating platform paradigm causes both reactive and anticipatory postural control strategies to be generated to overcome the same perturbation (Mills and Sveistrup, 2018).

While these reactive and anticipatory postural control strategies have been studied in children with other motor impairments (e.g., cerebral palsy; Mills et al., 2018), to our knowledge, they have not been studied in children with DCD during continuous dynamic movement. Additionally, no previous work has studied the structure of postural sway characteristics in children with DCD, nor evaluated the association with muscle activation and coordination. Therefore, the primary aim of this study was to compare postural responses to continuous platform oscillations between children with DCD and TD children. The secondary aim of this study was to determine if children with DCD were able to modify postural responses after prolonged exposure to platform movement. We hypothesized that children with DCD would be less able to adapt their postural responses compared to TD children after prolonged exposure to platform movement.

## 2. Materials and methods

### 2.1. Participants

Eighteen children with DCD and seven TD children participated in this study. Children with DCD were recruited through parental support groups on social media (e.g., Facebook). TD children were recruited via social media and convenience sampling (e.g., sibling of child with DCD). Participant characteristics are shown in Table 1. Children in the DCD group satisfied the Diagnostic and Statistical Manual of Mental Disorders (DSM-5) criteria (American Psychiatric Association, 2013), whereby they exhibit substandard motor ability, relative to their chronological age, since early development. Prior to data collection, parents/guardians completed the Developmental Coordination Disorder Questionnaire (Wilson et al., 2009) to confirm that their child had significant movement difficulties that interfered with balance, did not suffer from any general medical condition known to affect sensorimotor function, and had no diagnosed learning difficulties (DSM-5 criteria B, C, D). If any known medical conditions or learning difficulties were identified, these children were excluded from the study. Children with DCD were required to score ≤ 5^th^ percentile (overall), reflecting definite motor impairment (DSM-5 criteria A), and ≤ 15^th^ percentile (balance subscale), reflecting ‘risk’ of motor impairment, on the Movement Assessment Battery for Children, Second Edition (MABC-2; Henderson et al., 1992). TD children were required to score > 15^th^ percentile (balance subscale), reflecting no motor impairment. Parents/guardians also completed the Attentional Deficit Hyperactivity Disorder (ADHD) Rating Scale – VI (DuPaul et al., 1998). The institutional research ethics committee granted ethical approval. Written informed consent was obtained from parents/guardians and written assent given by children, in accordance with the Declaration of Helsinki.

**Table 1:** Mean ± standard deviation participant characteristics.

	DCD	TD
*N* (male/female)	18 (13/5)	7 (2/5)
Age (years)	9 ± 1	9 ± 1
Height (*m*)	1.41 ± 0.07	1.31 ± 0.09
Body Mass (kg)	38.9 ± 9.6	29.7 ± 12.4
MABC-2 Percentile (Overall)	2 ± 3	-
MABC-2 Percentile (Balance)	3 ± 3	56 ± 25
ADHD	90 ± 13	-

DCD, Children with developmental coordination disorder; TD, typically developing children; MABC-2, movement assessment battery for children.

### 2.2. Experimental protocol

**Figure 1:**
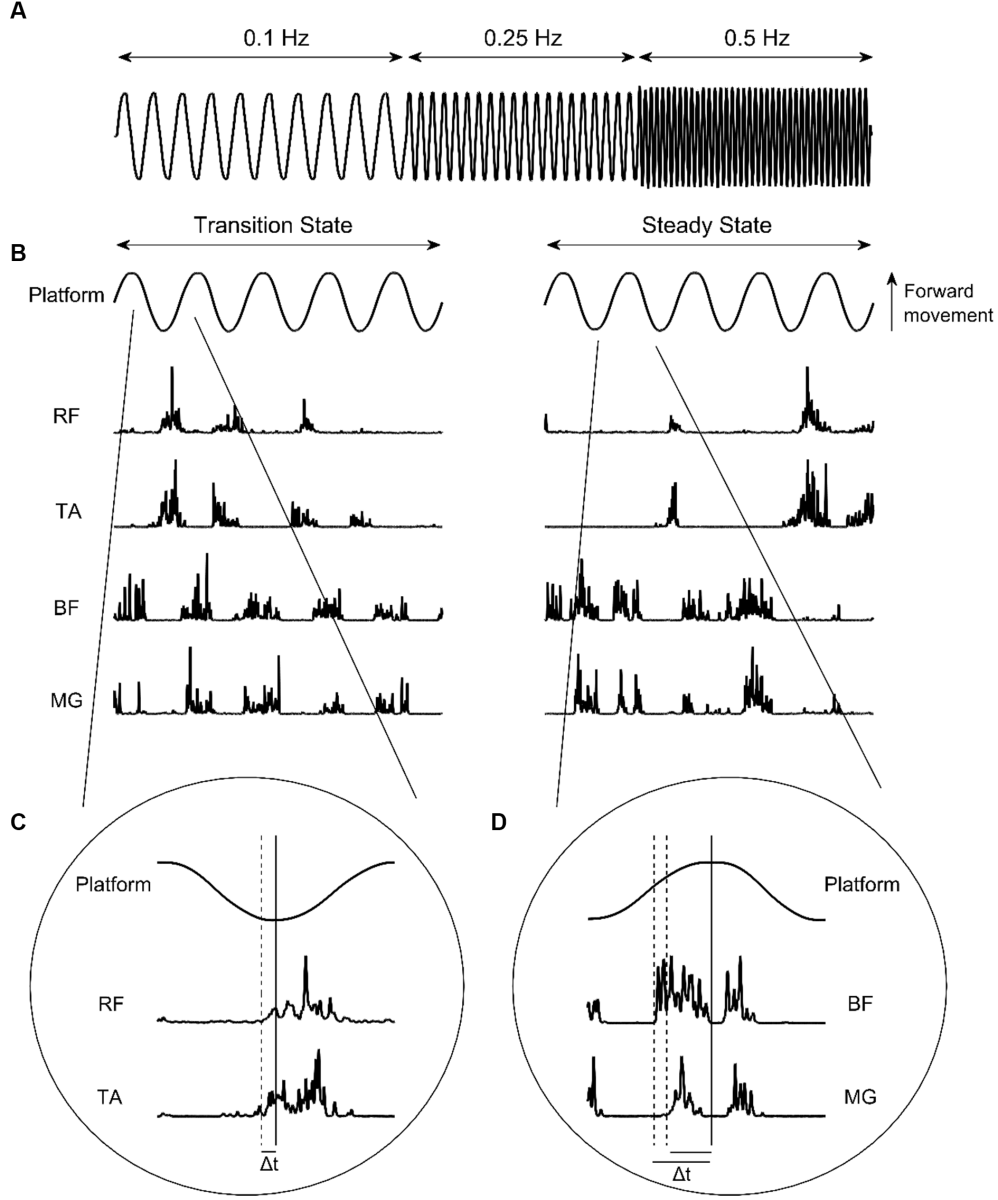
**(A)** Platform oscillation frequencies. **(B)** Platform oscillations at 0.5 Hz and corresponding EMG intensities from the rectus femoris (RF), tibialis anterior (TA), bicep femoris (BF), and medial gastrocnemius (MG) during transition-state and steady-state cycles. **(C)** Identification of anterior muscle activity onset. **(D)** Identification of posterior muscle activity onset. Solid vertical lines indicate platform change of direction. Dashed vertical lines indicate muscle activity onset. Δt indicates muscle onset latency.

The experimental protocol for this study was adapted from others described previously (Bugnariu and Sveistrup, 2006; Mills and Sveistrup, 2018). Participants stood upright with eyes open and feet shoulder width apart in the centre of a moveable platform. The platform was driven by electromagnetic propulsion, controlled via custom written software (Labview v19 SP1, National Instruments, Austin, Texas) through a DAQ card (USB-6210, National Instruments). Participants were instructed to maintain balance and avoid taking steps unless necessary. If steps were taken, participants were instructed to return to their initial position as quickly as possible. The platform translated 10 cm peak-to-peak in the antero-posterior direction. Two trials of ten sinusoidal oscillations at a frequency of 0.1 Hz, twenty oscillations at 0.25 Hz, and forty oscillations at 0.5 Hz (Figure 1A) were presented, with frequency changes presented sequentially and automatically. Participants were aware that platform frequency would increase, however they were not informed as to when this would occur.

Full body kinematics were collected at 100 Hz using a 10-camera motion analysis system (Qualisys v2021.1, Gothenburg, Sweden). Passive retro-reflective markers (*n* = 47) were positioned on all body segments (modified Plug-in Gait model). Two additional markers were positioned on the oscillating platform to record its position. For outcome measures described below, head and trunk angle, and whole-body COM were calculated in Visual 3D (v2021.06.2, C-Motion, Rockville, MD). Bilateral surface electromyography (EMG; Delsys Inc., Natick, United States) from rectus femoris (RF), biceps femoris (BF), tibialis anterior (TA), and medial gastrocnemius (MG) muscles were collected at 1000 Hz in Qualisys. Centre of pressure data were collected using a Kistler force plate (Type 9281B, Kistler Instrument Corp., Winterthur, Switzerland) at 1000 Hz. Force data were recorded in BioWare software (v5.4.3.0), synchronized to motion data by the Qualisys trigger.

### 2.3. Outcome measures

**Figure 2:**
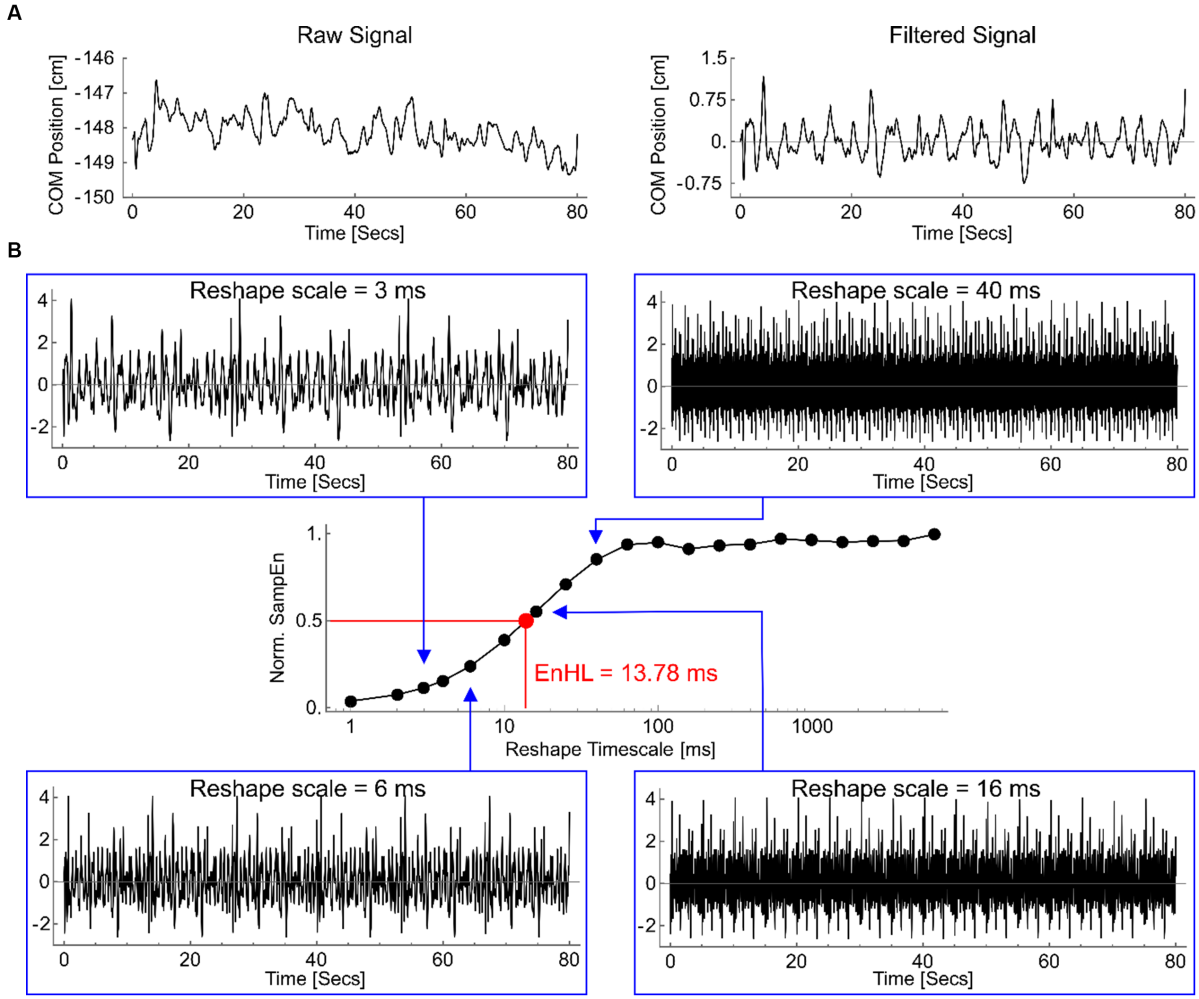
**(A)** An example medio-lateral COP displacement signal, from 0.25 Hz platform oscillation, as recorded (left) and after filtering (right). **(B)** Filtered signal reshaped at timescales of 3 ms (top left), 6 ms (lower left), 16 ms (lower right) and 40 ms (top right). Note the original repeating pattern of fluctuations is reduced as the reshape timescale increases. The normalized sample entropy values (SampEn) for each of these signals, and for all other reshape timescales, are shown in the central graph (log scale on *x*-axis). The timescale at which the normalized SampEn = 0.5 is highlighted (red), defining the EnHL for this signal as 13.78 ms.

At each platform frequency, the number of cycles containing a step were manually counted at the time of data collection and verified using motion capture data. Centre of pressure (COP) area was calculated using a 90% confidence ellipse. COM displacement variability in the antero-posterior and medio-lateral directions were assessed in terms of each signals standard deviation (SD) and the timescale over which short-term fluctuations in the signal persisted, calculated as the Entropy Halflife (EnHL). In the antero-posterior direction, both absolute and adjusted data are presented, whereby platform displacement was subtracted from COM data. To calculate EnHL, the COM in the antero-posterior and medio-lateral directions were split into equal length epochs containing all cycles within a single platform oscillation frequency. Each signal was high-pass filtered (2nd order Butterworth, 10 Hz cut-off) to attenuate temporal oscillations imposed by the platform movement (Figure 2A). The filtered signal was standardized (mean = 0, SD = 1) and a reshape timescale approach (Zandiyeh and Von Tscharner, 2013) used to generate restructured time series with increasing time intervals (1 ms – 6 s) between consecutive data points (Figure 2B). The sample entropy (SampEn) of each reshaped signal was calculated using a freely available software (Goldberger et al., 2000), with *m* = 1 and *r* = 0.2. SampEn provides the conditional probability that a time series of *m* data points remains affiliated, with a tolerance of *r*, if a data point is added to it (Richman and Moorman, 2000). Resulting SampEn values increase (indicating less regularity) as the reshape scale increases, reflecting the breakdown of short-term signal fluctuations (Figure 2B). The series of SampEn values produced were normalized to the maximum SampEn calculated for the original time series (when *m* = 0 and *r* = 0.2). This normalization means the reshape timescale at which SampEn = 0.5 represents the timescale at which the signal transitions from containing regular, structured fluctuations to being random (Zandiyeh and Von Tscharner, 2013) called the EnHL. These analyses were completed using custom written code in Wolfram Mathematica (version 11.1.1).

Head anchoring index (AI) was calculated using Eq. (1) (Mills and Sveistrup, 2018) to determine the stabilization strategy of the head in relation to both the global environment and the trunk segment:


(1) $$\mathrm{AI}=\left[\sigma_{r}^{2}-\sigma_{a}^{2}\right]÷\left[\sigma_{a}^{2}+\sigma_{r}^{2}\right]$$

where *σ*_a_ is the SD of the absolute head angle relative to the global coordinate system, and *σ*_r_ is the SD of the head relative to the trunk segment. A positive AI indicates a head-stabilized-in-space strategy. A negative AI indicates a head-stabilized-to-trunk strategy.

To calculate muscle activity onset latencies, EMG signals were decomposed into time-frequency space using an EMG specific wavelet analysis approach (Von Tscharner, 2000). Specifically, a filter bank of *k* = 11 non-linearly scaled wavelets with central frequencies spanning 6.90–395.44 Hz was used to resolve the EMG signal intensities into time/frequency space. Total intensity was calculated as the sum of the signal power contained within wavelets 1 ≥ *k* ≤ 10, providing a representation of the signal power at each time point whilst removing effects of low frequency signal components (i.e., contained within the first wavelet, *k* = 0).

The occurrence of muscle activity in respect to the relevant platform change of direction were identified manually using the *ginput* function in MATLAB (R2022a, MathWorks Inc., Natwick, MS, USA). To be considered for inclusion as muscle activity, EMG intensity had to meet or exceed two SDs above baseline (defined as the quiet period prior to trial start) and last for more than 50 ms (Mills and Sveistrup, 2018). For RF and TA, this was when the platform transitioned from backward to forward direction. For BF and MG, this was when the platform transitioned from forward to backward direction (Figures 1B–D). To remove subjectivity of this method, a custom MATLAB script was subsequently used. Firstly, the EMG intensities at the manually identified muscle activity onset times were determined, and averaged for each muscle to calculate an onset threshold. Activity onset times were then automatically adjusted using the script, so that all activity onsets for a specified participant occurred when EMG intensity surpassed their defined muscle threshold. Lastly, the total activity time of each muscle ‘burst’ was calculated as the time between activity onset, and the first subsequent instance that the EMG intensity envelope dropped below the onset threshold. All muscle activity data were expressed as a percentage of half-cycle time, to allow for comparisons between different platform frequencies. Muscle activity bursts were coded as anticipatory where they occurred before change of direction, and as reactive where they occurred after change of direction.

For AI and EMG data, platform frequencies were sub-divided into ‘transition-state’ and ‘steady-state’. Transition-state was defined as the first 3 cycles at 0.1 Hz, and the first 5 cycles at 0.25 and 0.5 Hz. Steady-state was defined as a period within the last half of each frequency that contained 5 cycles without stepping at 0.1 Hz, and a period of 8–10 cycles without stepping at 0.25 and 0.5 Hz, whereby the movement of the platform is predictable (Bugnariu and Sveistrup, 2006).

### 2.4. Statistical analysis

All statistical analyses were completed using RStudio (RStudio 1.3.959). Descriptive statistics (Table 1) are reported as mean ± standard deviation (SD). A linear-mixed model (LMM; lme4 package; Bates et al., 2015) was developed to quantify differences for each outcome measure (number of steps, COM SD, COM EnHL, COP area, head anchoring index, muscle onset latency and total excitation time) between groups (DCD vs. TD), platform frequencies (0.1 Hz vs. 0.25 Hz vs. 0.5 Hz) and platform state (transition vs. steady-state) (fixed effects). Participant ID was included as a random effect. Assumptions of linearity and normality distributions of the model were checked visually, and homogeneity of variance assessed using Levene’s Test (*p* > 0.05; Levene, 1960). Estimated means for each variable were derived from the model using the *emmeans* package, and are reported as mean ± standard error (SE). To identify between-group and between-state differences, Tukey’s pairwise comparisons were conducted. Statistical significance was set at *p* < 0.05. Effect sizes (ES) were also calculated using the *effsize* package, and considered trivial (< 0.2), small (≥ 0.2 to <0.6), moderate (≥ 0.6 to <1.2), large (≥ 1.2 to <2.0), or very large (≥ 2.0) (Batterham and Hopkins, 2006), and are presented as ES ± 90% confidence intervals. ES were considered unclear if the 90% confidence intervals included substantial positive and negative values (≥ ± 0.2; Hopkins et al., 2009).

## 3. Results

### 3.1. Stepping responses

One child with DCD took steps during 1 cycle at 0.1 Hz and 0.25 Hz. Three children with DCD took steps during 1 cycle at 0.25 Hz. No TD children took any steps at either 0.1 Hz or 0.25 Hz. At 0.5 Hz, 16 out of 18 children with DCD, and six out of seven TD children took steps throughout the trial. LMM estimated means showed that children with DCD took steps during more cycles to maintain balance than TD children at 0.5 Hz (8 ± 1 vs. 3 ± 2, large ES, 1.17 ± 0.47, *p* = 0.129), and at the other two frequencies (vs. 0.1 Hz, 8 ± 1 vs. 0 ± 1, large ES: 1.75 ± 0.35; *p* < 0.001; vs. 0.25 Hz, 8 ± 1 vs. 0 ± 1, large ES: 1.72 ± 0.35; *p* < 0.001).

### 3.2. COM variability

#### 3.2.1. COM standard deviation

**Figure 3:**
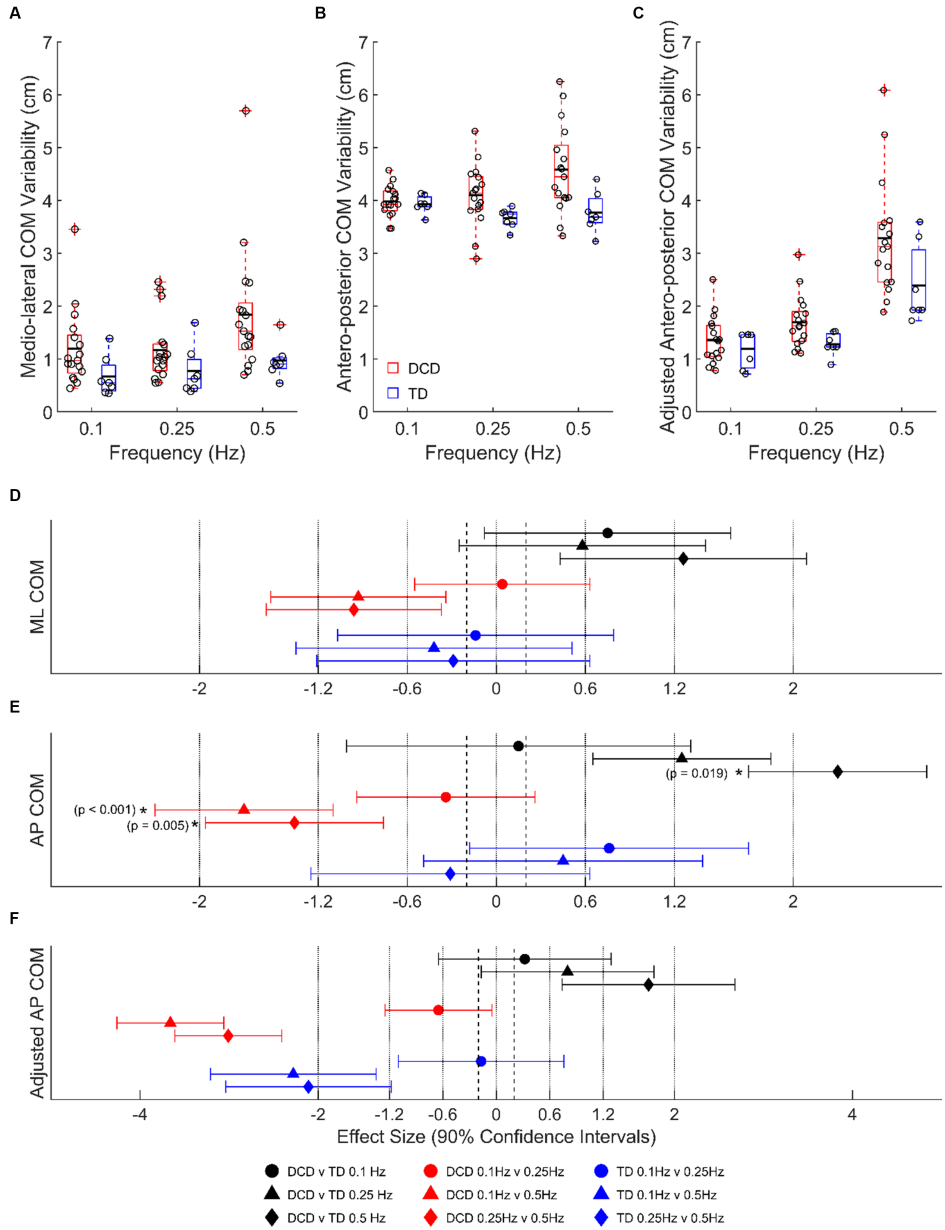
Linear-mixed model estimated centre of mass variability, based on signal standard deviation, in the **(A)** medio-lateral, **(B)** absolute antero-posterior, and **(C)** antero-posterior direction adjusted for platform movement. Solid horizontal black lines indicate group averages. Effect sizes with 90% confidence intervals from **(D)** medio-lateral, **(E)** absolute antero-posterior, and **(F)** adjusted antero-posterior centre of mass variability comparisons. Positive/negative effect sizes in **(D–F)** represent smaller/greater variability for 2nd comparator of each pairing. *Significant difference (*p* < 0.05). DCD, children with developmental coordination disorder; TD, typically developing children; ML, medio-lateral; AP, antero-posterior; COM, centre of mass.

Children with DCD had a greater LMM estimated COM SD, than TD children in the medio-lateral direction at 0.1 Hz (1.20 ± 0.19 vs. 0.67 ± 0.29 cm, moderate ES, 0.75 ± 0.83, *p* = 0.661) and 0.5 Hz (1.84 ± 0.19 vs. 0.97 ± 0.29 cm, large ES, 1.26 ± 0.83, *p* = 0.133), and antero-posterior direction at 0.25 Hz (4.10 ± 0.13 vs. 3.67 ± 0.20 cm, large ES, 1.25 ± 1.16, *p* = 0.465) and 0.5 Hz (4.58 ± 0.13 vs. 3.77 ± 0.20 cm, very large ES, 2.30 ± 1.16, *p* = 0.019) (Figure 3). In children with DCD, COM SD increased with task difficulty in both medio-lateral (moderate ESs: 0.93–0.96; *p* > 0.05) and antero-posterior (large ESs: 1.36–1.70; *p* < 0.01) directions (Figure 3), whereas there was no change in TD children (unclear ESs; *p* > 0.05). When platform displacement was accounted for in the antero-posterior direction, all observed differences between groups and platform frequencies were still present (Figures 3C,F).

#### 3.2.2. COM entropy halflife

**Figure 4:**
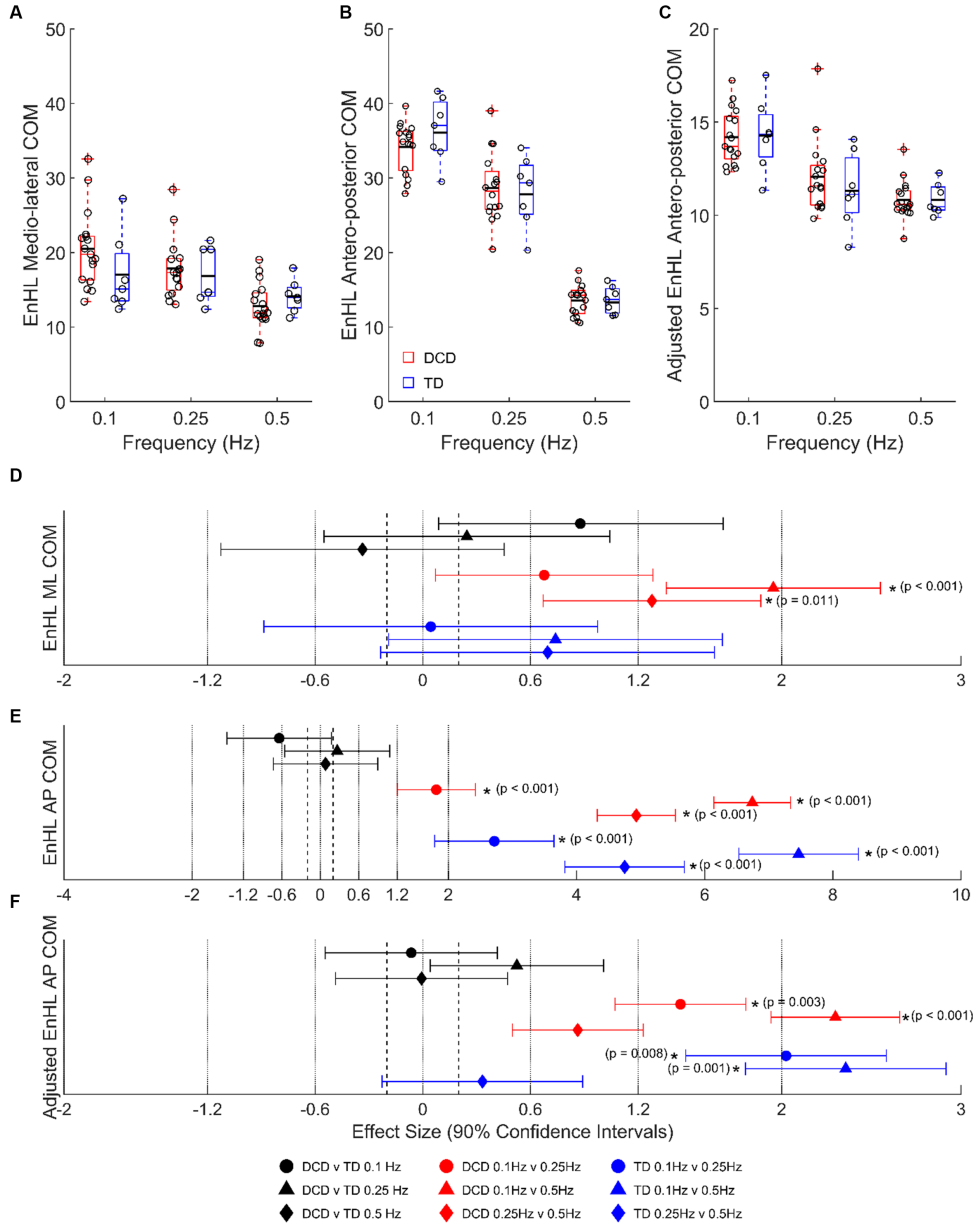
Linear-mixed model estimated centre of mass entropy halflife (EnHL; expressed here in milliseconds) in the **(A)** medio-lateral, **(B)** absolute antero-posterior, and **(C)** antero-posterior direction adjusted for platform movement. Solid horizontal black lines indicate group averages. Effect sizes with 90% confidence intervals from **(D)** medio-lateral, **(E)** absolute antero-posterior, and **(F)** adjusted anterior–posterior centre of mass EnHL comparisons. Positive/negative effect sizes in **(D−F)** represent shorter/longer EnHL for 2nd comparator of each pairing. *Significant difference (*p* < 0.05). DCD, children with developmental coordination disorder; TD, typically developing children; ML, medio-lateral; AP, antero-posterior; COM, centre of mass; EnHL, entropy halflife.

At 0.1 Hz, children with DCD had a longer LMM estimated COM EnHL in the medio-lateral direction (20.49 ± 0.99 vs. 17.04 ± 1.57 ms, moderate ES, 0.88 ± 0.79, *p* = 0.441), and a shorter COM EnHL in the antero-posterior direction (34.14 ± 0.86 vs. 36.10 ± 1.33 ms, moderate ES, 0.64 ± 0.81, *p* = 0.777) than TD children (Figure 4). In children with DCD, COM EnHL decreased with increased task difficulty in both medio-lateral (moderate [0.1 vs. 0.25 Hz, *p* = 0.438] to large [0.1 & 0.25 vs. 0.5 Hz, *p* < 0.05] ESs: 0.68–1.95) and antero-posterior directions (large to very large ESs: 1.81–6.74; *p* < 0.001). COM EnHL differences in the antero-posterior direction were still present when accounting for platform displacement (moderate to large ESs: 0.86–2.30; *p* < 0.05) (Figure 4C). In TD children, COM EnHL was similar regardless of platform frequency in the medio-lateral direction (Figure 4A), however COM EnHL decreased with increased task difficulty in the antero-posterior direction (very large ESs: 2.72–7.46; *p* < 0.001). When accounting for platform displacement, differences in 0.1 vs. 0.5 Hz (very large ES: 2.02 ± 0.93; *p* = 0.008) and 0.1 vs. 0.25 Hz (very large ES: 2.36 ± 0.93; *p* = 0.001) were still present in both groups (Figure 4C).

### 3.3. COP area

No difference in LMM estimated COP area was detected between groups at 0.1 Hz (46 ± 30 vs. 35 ± 39 cm^2^, unclear ES, 0.12 ± 0.95, *p* = 0.999) and 0. 25 Hz (51 ± 30 vs. 46 ± 39 cm^2^, unclear ES, 0.05 ± 0.95, *p* = 0.999), however children with DCD had a greater COP area than TD children at 0.5 Hz (250 ± 30 vs. 113 ± 39 cm^2^, large ES, 1.60 ± 0.95, *p* = 0.069). COP area increased with task difficulty in both children with DCD (very large ESs: 2.33–2.39; *p* < 0.001) and TD children (moderate ESs: 0.78–0.91; *p* > 0.05).

### 3.4. Anchoring index

**Figure 5:**
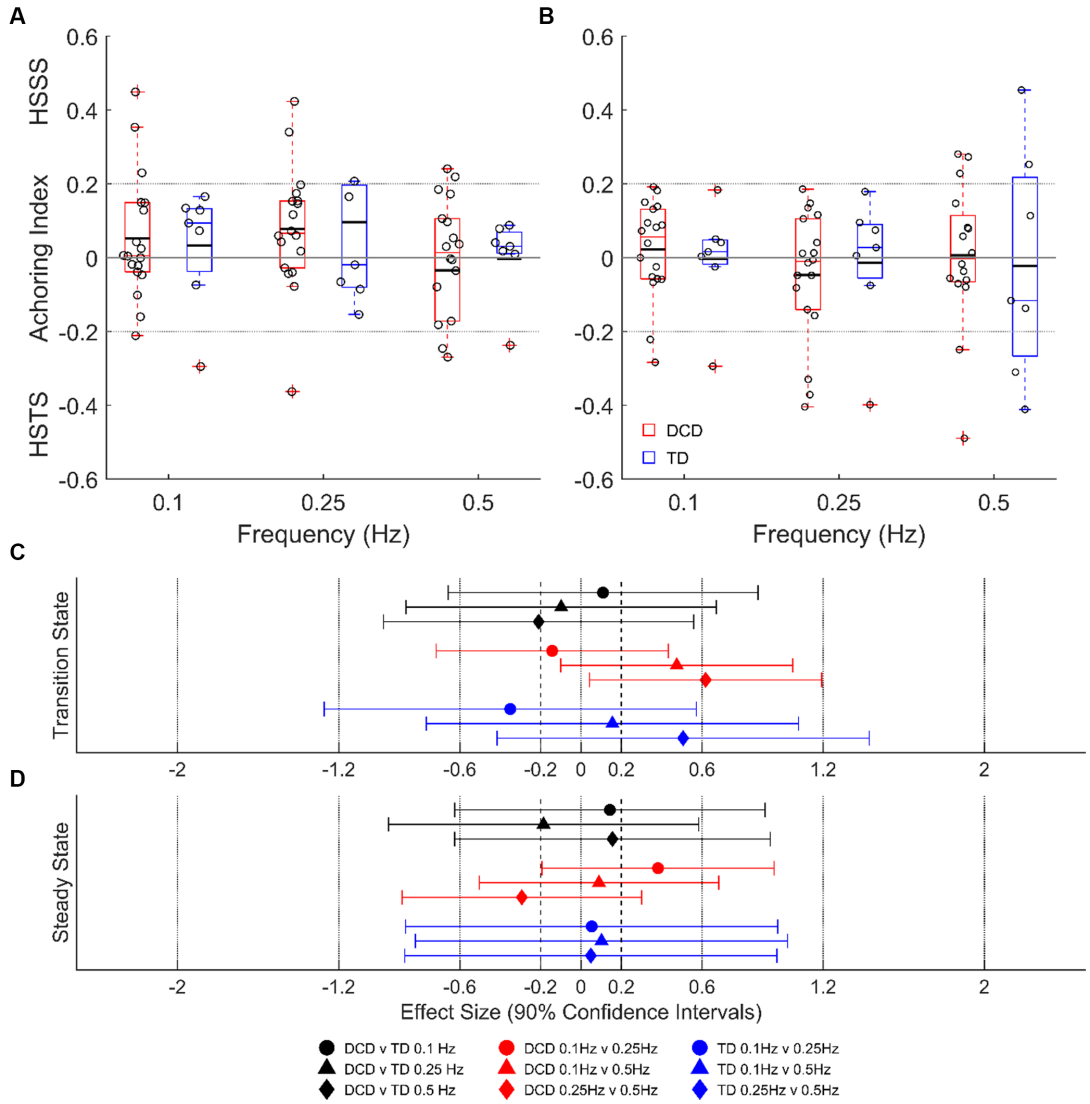
Linear-mixed model estimated head anchoring index during transition-state **(A)** and steady state **(B)** cycles. Dashed lines at ±0.2 indicate the threshold for a given strategy. Effect sizes with 90% confidence intervals from transition-state **(C)** and steady-state **(D)** cycles. DCD, children with developmental coordination disorder; TD, typically developing children; HSSS, head stabilised in space strategy; HSTS, head stabilised to trunk strategy.

Despite some individual participants adopting a head-stabilized-in-space strategy or head-stabilized-to-trunk strategy, average data indicate no clear head stabilization strategy in either group (AI of <−0.2 or > 0.2; Figure 5). There were no group differences detected during transition or steady-state cycles at any platform frequency (unclear ESs; *p* > 0.05), and no state differences detected in either group (unclear ESs; *p* > 0.05).

### 3.5. Muscle activity

LMM estimated muscle activity data for transition-state and steady-state cycles are shown in Table 2. In general, both groups tended to activate their muscles earlier and for longer as task difficulty increased. At 0.25 Hz, muscle excitation occurred earlier in children with DCD in the RF (moderate ES: 1.08 ± 1.07), TA (large ES: 1.62 ± 1.32) and MG (large ES: 1.49 ± 0.85) during transition-state cycles, and in the MG (moderate ES: 1.07 ± 0.85) during steady-state cycles than TD children. Muscle excitation duration of the MG was longer in children with DCD (moderate ES: 0.93 ± 1.03) than TD children during steady-state cycles. At 0.5 Hz, muscle excitation of the MG occurred earlier in children with DCD during both transition-state (moderate ES: 1.13 ± 0.79) and steady-state cycles (large ES: 1.90 ± 0.81), and for longer in the BF (moderate ES: 1.02 ± 1.04) and MG (large ES: 1.58 ± 0.92) during transition-state cycles, and in the BF (moderate ES: 1.05 ± 1.04) and MG (large ES: 1.31 ± 0.94) during steady-state cycles than TD children.

**Table 2:** Linear-mixed model estimated mean ± standard error timing of muscle activity during transition-state and steady-state cycles.

		Transition-state				Steady-state			
		**Onset latency (%)**		**Total excitation time (%)**		**Onset latency (%)**		**Total excitation time (%)**	
		**DCD**	**TD**	**DCD**	**TD**	**DCD**	**TD**	**DCD**	**TD**
**0.1 Hz**	**RF**	9.13 ± 3.72	9.79 ± 4.47	7.03 ± 1.93	2.39 ± 2.44^***^	3.82 ± 3.03	4.33 ± 4.09	10.07 ± 1.67^††^	9.71 ± 2.31^††††^
	**TA**	−1.40 ± 4.45	4.45 ± 5.75	7.02 ± 1.51	5.12 ± 1.95	7.08 ± 3.69^††^	2.84 ± 6.25	5.96 ± 1.25	3.71 ± 2.12
	**BF**	5.51 ± 5.98	−10.81 ± 10.69	6.87 ± 2.79	13.53 ± 4.99	0.81 ± 5.98	−15.11 ± 7.94^**^	6.29 ± 2.79	2.47 ± 3.71^†††^
	**MG**	−4.67 ± 3.21	−13.28 ± 5.43^**^	5.90 ± 1.75	7.59 ± 2.95	−2.80 ± 3.21	−0.57 ± 5.01^††^	8.73 ± 1.75^†^	7.02 ± 2.72
**0.25 Hz**	**RF**	−8.64 ± 5.58	8.13 ± 8.21^**^	21.06 ± 4.74	14.77 ± 6.92	−3.68 ± 5.19	8.66 ± 8.21	17.34 ± 4.38	26.38 ± 6.92
	**TA**	−10.81 ± 4.00	4.44 ± 6.17^***^	10.09 ± 1.78	9.89 ± 2.71	−6.62 ± 3.85^†^	−1.16 ± 6.17	12.66 ± 1.69^†^	9.02 ± 2.71
	**BF**	−16.98 ± 3.88	−10.62 ± 5.89	15.19 ± 2.59	12.92 ± 3.91	−13.82 ± 3.74	−13.01 ± 6.49	16.51 ± 2.49	12.84 ± 4.33
	**MG**	−20.64 ± 2.16	−8.58 ± 3.46^***^	12.41 ± 1.58	11.17 ± 2.53	−22.27 ± 2.16	−13.63 ± 3.46^**^	14.98 ± 1.58^†^	10.43 ± 2.54^**^
**0.5 Hz**	**RF**	−17.08 ± 5.46	−3.95 ± 9.23	33.64 ± 7.90	30.84 ± 13.36	−15.69 ± 5.84	−18.43 ± 8.48	42.70 ± 8.46	25.69 ± 12.27
	**TA**	−19.84 ± 4.24	−17.07 ± 6.80	25.89 ± 3.29	19.97 ± 5.28	−19.18 ± 4.65	−20.74 ± 6.80	18.85 ± 3.44^††^	23.34 ± 5.28
	**BF**	−21.19 ± 3.30	−13.50 ± 4.91	39.64 ± 7.08	18.90 ± 10.43^**^	−20.32 ± 3.22	−16.11 ± 4.91	44.40 ± 7.08	23.10 ± 10.43^**^
	**MG**	−25.98 ± 2.06	−16.69 ± 3.31^**^	32.16 ± 3.25	14.47 ± 5.21^***^	−29.67 ± 2.20^†^	−14.01 ± 3.31^***^	29.89 ± 3.45	15.25 ± 5.21^***^

Negative onset latencies indicate muscle excitation occurred before platform change of direction.

^*^Small, ^**^moderate or ^***^large effect size difference between DCD and TD.

^†^Small, ^††^moderate, ^†††^large, or ^††††^very large effect size difference between transition-state and steady-state cycles.

RF, rectus femoris; TA, tibialis anterior; BF, bicep femoris; MG, medial gastrocnemius; DCD, children with developmental coordination disorder; TD, typically developing children.

At 0.25 Hz, children with DCD generally activated their muscles at a similar time between platform states (except TA excitation occurred later in steady-state), however excitation duration was longer in steady-state cycles for the TA (small ES: 0.49 ± 0.61) and GM (small ES: 0.52 ± 0.58) than in transition-state cycles. During steady-state cycles, TD children tended to activate their muscles earlier and for shorter durations than in transition-state cycles, however all effect sizes were unclear. At 0.5 Hz, no clear trends were observed in muscle excitation onset time or excitation duration between platform states in either group. Full ES comparisons can be found in Supplementary Figures S1, S2.

## 4. Discussion

This study is the first to assess the postural and neuromuscular responses of children with DCD using a continuous balance perturbation paradigm. As expected, children with DCD were generally more unstable than TD children, particularly at the highest platform frequency. An increase in the number of children who took steps at 0.5 Hz reflects the increased difficulty of the task for both groups (Streepey and Angulo-Kinzler, 2002). However, children with DCD took steps more often than TD children to maintain balance (large ES). Children with DCD also had a greater COM variability (SD) than TD children in both the antero-posterior (large to very large ESs) and medio-lateral (moderate to large ESs) directions (Figure 3), indicating greater postural sway. This was further supported by the greater COP area covered by children with DCD (large ES).

At the fastest platform frequencies, children with DCD tended to adopt a different muscle excitation strategy to TD children. Activating their muscles earlier and for longer may suggest that children with DCD attempt to predict and react to postural disturbances, however the resulting anticipatory muscle excitation patterns do not seem as finely tuned to the perturbation as those demonstrated by TD children. Additionally, children with DCD made more, non-structured (random) postural adjustments in the medio-lateral direction as task difficulty increased. Therefore, data from the current study indicate an altered neuromuscular coordination in children with DCD, which should be considered in future training interventions to improve balance control.

Despite the reduced stability of children with DCD, there was no detected difference in their global stabilization strategy compared to TD children. Children with DCD showed no preference for either a head-stabilized-to-trunk strategy, or a head-stabilized-in-space strategy (Figure 5), whereas other populations with known balance deficits, such as children with cerebral palsy (Mills et al., 2018) and adults with Parkinson’s disease (Mesure et al., 1999), adopt a head-stabilized-to-trunk strategy. This may be explained by a poor organization of body movements in relation to the global environment, often associated with DCD (Green and Payne, 2018).

Children with DCD did however, adopt a different neuromuscular strategy to TD children at the faster platform frequencies. Generally, the organization of muscle excitation was distal to proximal in children with DCD, indicating an ankle strategy was implemented to maintain balance (Massion, 1994). Whilst this was also the case for the anterior muscles of TD children, there were some instances whereby average posterior muscle excitation was ordered proximal to distal (Table 2). This may indicate that TD children were able to switch between an ankle and hip strategy to maintain balance (Horak and Nashner, 1986). Children with DCD tended to activate their muscles earlier and for longer than TD children, regardless of platform state (Table 2). Whilst this does suggest that children with DCD attempt to predict and react to postural disturbances (Cordo and Nashner, 1982), the resultant anticipatory muscle excitations are different to those demonstrated by TD children. Thus, a lack of appropriate muscular reactions to balance perturbations may explain poor dynamic balance control in children with DCD.

Previous work has shown that children with DCD do not make postural adaptations when exposed to repeated discrete perturbations (Cheng et al., 2022). During our continuous perturbations, neither group made postural adjustments with prior knowledge of platform movement at the fastest platform frequency, as both muscle excitation onset time and total excitation duration remained similar between transition-state and steady-state cycles. However, this likely reflects the increased difficulty of the task at 0.5 Hz, as TD children were able to make postural adjustments with prior knowledge of platform movement at 0.25 Hz (Table 2). At 0.25 Hz, TD children activated their muscles earlier and for a shorter duration during steady-state cycles, which may suggest that they were able to better anticipate platform movement compared to transition-state cycles. In contrast, there were no changes in muscle excitation onset times between platform states in children with DCD, and muscle excitation duration was indeed longer in steady-state cycles. Overall, data from the current study indicate an altered neuromuscular coordination in children with DCD, which should be considered in future training interventions to improve balance control.

While children with DCD exhibited greater postural sway than TD children (Figure 3), the structural organization of the antero-posterior COM variability (EnHL) did not differ between groups (Figure 4). This suggests that to maintain balance, the control strategies adopted by children with DCD resulted in a similar temporal organization of the antero-posterior COM movement as TD children, possibly explaining the similarity in the global kinematic outcome measures described above. However, surprisingly, the EnHL of the medio-lateral displacement of children with DCD became shorter as platform difficulty increased, whereas there was no change in TD children. This suggests that children with DCD made more, non-structured (random), postural adjustments in a plane orthogonal to platform movement as task difficulty increased. Previous work has shown those with DCD to explore more action space during a defined task by increasing available degrees of freedom (Golenia et al., 2018). Therefore, this increased, less structured variability in the medio-lateral plane, may be a compensatory mechanism as a result of the way children with DCD manage the degrees of freedom problem (Latash et al., 2007). It may also be explained by a lack of stiffening and/or appropriately organized recruitment of hip ab/adductor muscles, which are important for medio-lateral stability (Winter et al., 1996). However, as we did not measure muscle activity in these muscles, further work is required to confirm or deny this notion.

Some limitations should be acknowledged. Firstly, our sample size is small and does not include an even distribution of male/female participants. While sex differences in postural control have been shown previously in TD children (Smith et al., 2012), exploring sex differences between and within children with DCD and TD children was outside the scope of the current manuscript. Furthermore, it was not possible to accurately explore sex differences due to the insufficient number of data per sub-level (e.g., TD male participants, *n* = 2). Future work with larger sample sizes is needed. EMG data were only collected for eight lower limb muscles, yet conclusions are generalized to whole-body postural control. Further, our assumption that postural movement in the antero-posterior direction would be solely controlled by flexor/extensor muscles meant that all eight muscles considered for analysis were flexor/extensor muscles. Future work should therefore consider collecting EMG data from more muscles, and consider the role that ab/adductor and rotational muscles may play in ensuring postural stability in the antero-posterior direction. Future work should also consider assessing the EnHL of EMG data, to identify whether there are any differences in the temporal organization of muscle activity.

To conclude, data from the current study indicate that while children with DCD were not able to perform the task as well as TD children (more unstable), they were able to complete the task, actively working toward making similar global postural adjustments as TD children. However, to achieve a similar global stabilization strategy, children with DCD generated this response with a different neuromuscular strategy, activating their muscles earlier and for longer than TD children. Children with DCD also made more, non-structured, movements in a plane orthogonal to platform displacement as task difficulty increased, suggesting they utilize more degrees of freedom to overcome balance perturbations than TD children. Future work should examine the impact of balance training interventions on the muscle excitation patterns and coordination strategies of children with DCD, to ensure that appropriate interventions to improve balance can be prescribed. Future work should also consider the role of attentional deficits of children with DCD on postural control during continuous balance perturbations.

## Data availability statement

The raw data supporting the conclusions of this article will be made available by the authors, without undue reservation.

## Ethics statement

The studies involving humans were approved by the Faculty of Science and Engineering Research and Ethics Governance. The studies were conducted in accordance with the local legislation and institutional requirements. Written informed consent for participation in this study was provided by the participants’ legal guardians/next of kin.

## Author contributions

CH-A: Data curation, Formal analysis, Writing – original draft. EH-T: Conceptualization, Funding acquisition, Methodology, Supervision, Writing – review & editing. GW: Conceptualization, Funding acquisition, Methodology, Supervision, Writing – review & editing. RM: Conceptualization, Funding acquisition, Methodology, Project administration, Supervision, Writing – review & editing.

## References

[ref1] Adkin A. L. Frank J. S. Carpenter M. G. Peysar G. W. (2000). Postural control is scaled to level of postural threat. Gait Posture 12, 87–93. doi: 10.1016/S0966-6362(00)00057-6, PMID: 10998604

[ref2] American Psychiatric Association (2013). “Diagnostic and statistical manual of mental disorders DSM-5” in Jama the journal of the American Medical Association. 5th ed (Washington, DC: American Psychiatric Association)

[ref3] Bates D. Mächler M. Bolker B. M. Walker S. C. (2015). Fitting linear mixed-effects models using LME 4. J. Stat. Softw. 67, 1–48. doi: 10.18637/jss.v067.i01

[ref4] Batterham A. M. Hopkins W. G. (2006). Making meaningful inferences about magnitudes. Int. J. Sports Physiol. Perform. 1, 50–57. doi: 10.1123/ijspp.1.1.50, PMID: 19114737

[ref5] Bolton D. A. E. (2015). The role of the cerebral cortex in postural responses to externally induced perturbations. Neurosci. Biobehav. Rev. 57, 142–155. doi: 10.1016/j.neubiorev.2015.08.014 26321589

[ref6] Bugnariu N. Sveistrup H. (2006). Age-related changes in postural responses to externally- and self-triggered continuous perturbations. Arch. Gerontol. Geriatr. 42, 73–89. doi: 10.1016/j.archger.2005.05.003, PMID: 16084609

[ref7] Cheng Y. T. Chung L. M. Chung J. W. Schooling C. M. Gao Y. Bae Y. H.. (2022). Atypical adaptive postural responses in children with developmental coordination disorder: implications for rehabilitation. Gait Posture 98, 141–145. doi: 10.1016/j.gaitpost.2022.09.007, PMID: 36122429

[ref8] Cheng Y. T. Y. Tsang W. W. N. Schooling C. M. Fong S. S. M. (2018). Reactive balance performance and neuromuscular and cognitive responses to unpredictable balance perturbations in children with developmental coordination disorder. Gait Posture 62, 20–26. doi: 10.1016/j.gaitpost.2018.02.025, PMID: 29501972

[ref9] Cordo P. J. Nashner L. M. (1982). Properties of postural adjustments associated with rapid arm movements. J. Neurophysiol. 47, 287–302. doi: 10.1152/jn.1982.47.2.287, PMID: 7062101

[ref10] DuPaul G. J. Power T. J. Anastopoulos A. D. Reid R. (1998). ADHD rating scale—IV: checklists, norms, and clinical interpretation. New York: Guilford. doi: 10.1177/0734282905285792

[ref11] Ferrari E. Cooper G. Reeves N. D. Hodson-Tole E. F. (2020). Intrinsic foot muscles act to stabilise the foot when greater fluctuations in Centre of pressure movement result from increased postural balance challenge. Gait Posture 79, 229–233. doi: 10.1016/j.gaitpost.2020.03.011, PMID: 32446178

[ref12] Fong S. S. M. Lee V. Y. L. Chan N. N. C. Chan R. S. H. Chak W. K. Pang M. Y. C. (2011). Motor ability and weight status are determinants of out-of-school activity participation for children with developmental coordination disorder. Res. Dev. Disabil. 32, 2614–2623. doi: 10.1016/j.ridd.2011.06.013, PMID: 21767931

[ref13] Fong S. S. M. Ng S. S. M. Guo X. Wang Y. Chung R. C. K. Ki W. Y.. (2015). Deficits in lower limb muscle reflex contraction latency and peak force are associated with impairments in postural control and gross motor skills of children with developmental coordination disorder: a cross-sectional study. Medicine, Balt 94:e1785. doi: 10.1097/MD.0000000000001785, PMID: 26469921 PMC4616810

[ref14] Geuze R. H. Jongmans M. J. Schoemaker M. M. Smits-Engelsman B. C. M. (2001). Clinical and research diagnostic criteria for developmental coordination disorder: a review and discussion. Hum. Mov. Sci. 20, 1–5. doi: 10.1016/S0167-9457(01)00027-6 11471398

[ref15] Goldberger A. L. Amaral L. A. Glass L. Hausdorff J. M. Ivanov P. C. Mark R. G.. (2000). Physio Bank, physio toolkit, and physio net: components of a new research resource for complex physiologic signals. Circulation 101, E215–E220. doi: 10.1161/01.cir.101.23.e215, PMID: 10851218

[ref16] Golenia L. Bongers R. M. van Hoorn J. F. Otten E. Mouton L. J. Schoemaker M. M. (2018). Variability in coordination patterns in children with developmental coordination disorder (DCD). Hum. Mov. Sci. 60, 202–213. doi: 10.1016/j.humov.2018.06.009, PMID: 29957424

[ref17] Green D. Payne S. (2018). Understanding organisational ability and self-regulation in children with developmental coordination disorder. Curr. Dev. Disord. Rep. 5, 34–42. doi: 10.1007/s40474-018-0129-2 29497596 PMC5818572

[ref18] Haddad J. M. Rietdyk S. Claxton L. J. Huber J. (2013). Task-dependent postural control throughout the lifespan. Exerc. Sport Sci. Rev. 41, 123–132. doi: 10.1097/JES.0b013e3182877cc8 23364347 PMC3608710

[ref19] Hausdorff J. M. (2007). Gait dynamics, fractals and falls: finding meaning in the stride-to-stride fluctuations of human walking. Hum. Mov. Sci. 26, 555–589. doi: 10.1016/j.humov.2007.05.003, PMID: 17618701 PMC2267927

[ref20] Hausdorff J. M. Zemany L. Peng C. K. Goldberger A. L. (1999). Maturation of gait dynamics: stride-to-stride variability and its temporal organization in children. J. Appl. Physiol. 86, 1040–1047. doi: 10.1152/jappl.1999.86.3.1040, PMID: 10066721

[ref21] Henderson S. E. Sugden D. Barnett A. L. (1992). “Movement assessment battery for children-2” in Research in developmental disabilities. APA PsycTests. doi: 10.1037/t55281-000

[ref22] Hodson-Tole E. F. Wakeling J. M. (2017). Movement complexity and neuromechanical factors affect the entropic half-life of myoelectric signals. Front. Physiol. 8:679. doi: 10.3389/fphys.2017.00679, PMID: 28974932 PMC5610701

[ref23] Hopkins W. G. Marshall S. W. Batterham A. M. Hanin J. (2009). Progressive statistics for studies in sports medicine and exercise science. In. Med. Sci. Sports Exerc. 41, 3–12. doi: 10.1249/MSS.0b013e31818cb278 19092709

[ref24] Horak F. B. Nashner L. M. (1986). Central programming of postural movements: adaptation to altered support-surface configurations. J. Neurophysiol. 55, 1369–1381. doi: 10.1152/jn.1986.55.6.1369, PMID: 3734861

[ref25] Horak F. B. Wrisley D. M. Frank J. (2009). The balance evaluation systems test (BESTest) to differentiate balance deficits. Phys. Ther. 89, 484–498. doi: 10.2522/ptj.20080071, PMID: 19329772 PMC2676433

[ref26] Huxham F. E. Goldie P. A. Patla A. E. (2001). Theoretical considerations in balance assessment. Aust. J. Physiother. 47, 89–100. doi: 10.1016/S0004-9514(14)60300-7 11552864

[ref27] Kane K. Barden J. (2012). Contributions of trunk muscles to anticipatory postural control in children with and without developmental coordination disorder. Hum. Mov. Sci. 31, 707–720. doi: 10.1016/j.humov.2011.08.004, PMID: 21982786

[ref28] Latash M. L. Scholz J. P. Schöner G. (2007). Toward a new theory of motor synergies. Mot. Control. 11, 276–308. doi: 10.1123/mcj.11.3.276, PMID: 17715460

[ref29] Levene H. (1960). Robust tests for equality of variances. in Contributions to Probability and Statistics. ed. Olkin I. (Palo Alto, Calif: Stanford University Press) 69, 278–92.

[ref30] Massion J. (1994). Postural control system. Curr. Opin. Neurobiol. 4, 877–887. doi: 10.1016/0959-4388(94)90137-6 7888772

[ref31] Mesure S. Azulay J. P. Pouget J. Amblard B. (1999). Strategies of segmental stabilization during gait in Parkinson’s disease. Exp. Brain Res. 129, 573–581. doi: 10.1007/s002210050927 10638431

[ref32] Mills R. Levac D. Sveistrup H. (2018). Kinematics and postural muscular activity during continuous oscillating platform movement in children and adolescents with cerebral palsy. Gait Posture 66, 13–20. doi: 10.1016/j.gaitpost.2018.08.002, PMID: 30138742

[ref33] Mills R. S. Sveistrup H. (2018). Kinematics and postural muscular activity during continuous oscillating platform movement in children and adolescents. Exp. Brain Res. 236, 1479–1490. doi: 10.1007/s00221-018-5228-0, PMID: 29550878

[ref34] Richman J. S. Moorman J. R. (2000). Physiological time-series analysis using approximate entropy and sample entropy maturity in premature infants physiological time-series analysis using approximate entropy and sample entropy. Am. J. Phys. Heart Circ. Phys. 278, H2039–H2049. doi: 10.1152/ajpheart.2000.278.6.H2039 10843903

[ref35] Scott-Roberts S. Purcell C. (2018). Understanding the functional mobility of adults with developmental coordination disorder (DCD) through the international classification of functioning (ICF). Curr. Dev. Disord. Rep. 5, 26–33. doi: 10.1007/s40474-018-0128-3 29497595 PMC5818573

[ref36] Smith A. W. Ulmer F. F. Wong D. P. (2012). Gender differences in postural stability among children. J. Hum. Kinet. 33, 25–32. doi: 10.2478/v10078-012-0041-5, PMID: 23487417 PMC3588681

[ref37] Streepey J. W. Angulo-Kinzler R. M. (2002). The role of task difficulty in the control of dynamic balance in children and adults. Hum. Mov. Sci. 21, 423–438. doi: 10.1016/S0167-9457(02)00104-5, PMID: 12450677

[ref38] Turnock M. J. E. Layne C. S. (2010). Variations in linear and nonlinear postural measurements under Achilles tendon vibration and unstable support-surface conditions. J. Mot. Behav. 42, 61–69. doi: 10.1080/00222890903397103, PMID: 20018587

[ref39] Von Tscharner V. (2000). Intensity analysis in time-frequency space of surface myoelectric signals by wavelets of specified resolution. J. Electromyogr. Kinesiol. 10, 433–445. doi: 10.1016/S1050-6411(00)00030-4, PMID: 11102846

[ref40] Wakeling J. M. Hodson-Tole E. F. (2018). How do the mechanical demands of cycling affect the information content of the EMG? Med. Sci. Sports Exerc. 50, 2518–2525. doi: 10.1249/MSS.0000000000001713, PMID: 29975298

[ref41] Wilson B. N. Crawford S. G. Green D. Roberts G. Aylott A. Kaplan B. J. (2009). Psychometric properties of the revised developmental coordination disorder questionnaire. Phys. Occup. Ther. Pediatr. 29, 182–202. doi: 10.1080/01942630902784761, PMID: 19401931

[ref42] Winter D. A. Prince F. Frank J. S. Powell C. Zabjek K. F. (1996). Unified theory regarding a/P and M/L balance in quiet stance. J. Neurophysiol. 75, 2334–2343. doi: 10.1152/jn.1996.75.6.2334, PMID: 8793746

[ref43] Yam T. T. T. Fong S. S. M. (2019). Y-balance test performance and leg muscle activations of children with developmental coordination disorder. J. Mot. Behav. 51, 385–393. doi: 10.1080/00222895.2018.1485011, PMID: 30095371

[ref44] Zandiyeh P. Von Tscharner V. (2013). Reshape scale method: a novel multi scale entropic analysis approach. Physica A 392, 6265–6272. doi: 10.1016/j.physa.2013.08.023

[ref45] Zwicker J. G. Missiuna C. Harris S. R. Boyd L. A. (2012). Developmental coordination disorder: a review and update. Eur. J. Paediatr. Neurol. 16, 573–581. doi: 10.1016/j.ejpn.2012.05.005 22705270

