# Supplementary material for: Children with developmental coordination disorder are less able to fine-tune muscle activity in anticipation of postural perturbations than typically developing counterparts
Source: Front Hum Neurosci. 2023 Oct 27;17:1267424. doi: 10.3389/fnhum.2023.1267424 (PMC10641443; doi:10.3389/fnhum.2023.1267424)
Supplement: Supplementary file 1 [file Data_Sheet_1.PDF]

Supplementary Material

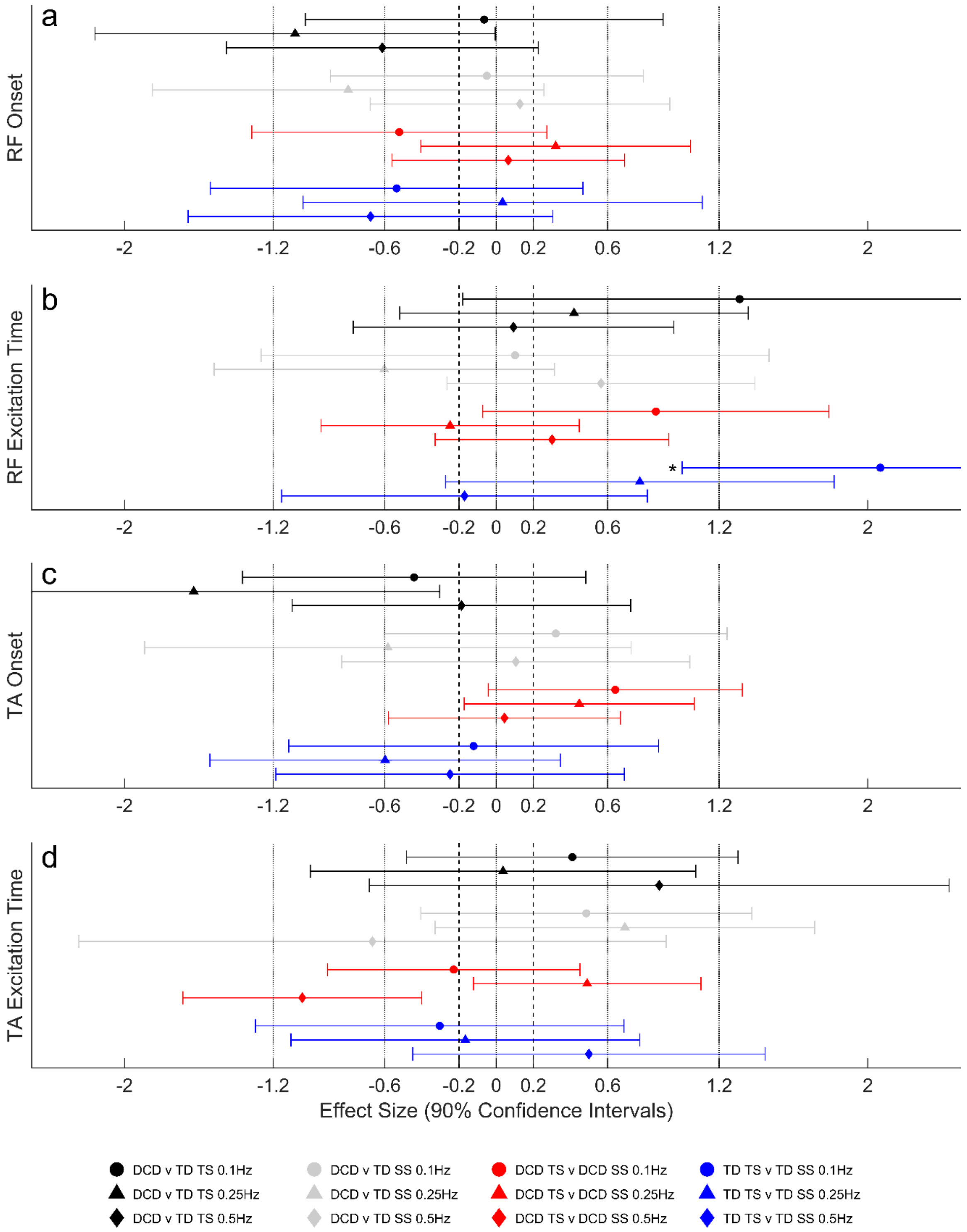

*Figure SM1. Effect sizes with 90% confidence intervals from rectus femoris onset latency (a) and total excitation time (b), and tibialis anterior onset latency (c) and total excitation time (d). *Significant difference ($p < 0.05$). DCD, children with developmental coordination disorder; TD, typically developing children; TS, transition-state; SS, steady-state; RF, rectus femoris; TA, tibialis anterior.*

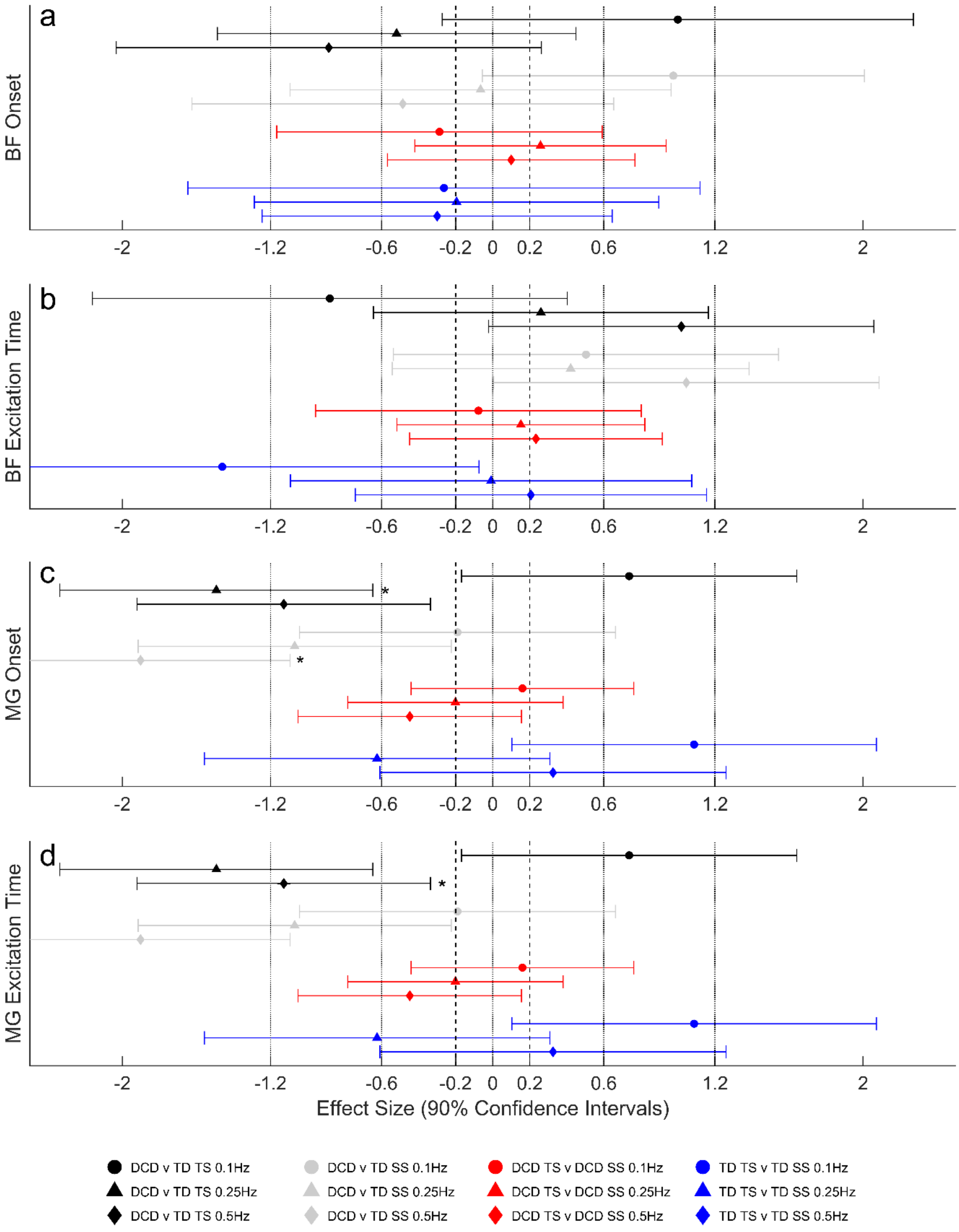

*Figure SM2. Effect sizes with 90% confidence intervals from bicep femoris onset latency (a) and total excitation time (b), and medial gastrocnemius onset latency (c) and total excitation time (d). *Significant difference ($p < 0.05$). **Significant different ($p < 0.01$). DCD, children with developmental coordination disorder; TD, typically developing children; TS, transition-state; SS, steady-state; BF, bicep femoris; MG, medial gastrocnemius.*
